# Experiences from a multimodal rhythm and music-based rehabilitation program in late phase of stroke recovery – A qualitative study

**DOI:** 10.1371/journal.pone.0204215

**Published:** 2018-09-18

**Authors:** Petra Pohl, Gunnel Carlsson, Lina Bunketorp Käll, Michael Nilsson, Christian Blomstrand

**Affiliations:** 1 Department of Clinical Neuroscience, Institute of Neuroscience and Physiology, Sahlgrenska Academy at University of Gothenburg, Gothenburg, Sweden; 2 Department of Activity and Health, and Department of Medical and Health Sciences, Linköping University, Linköping, Sweden; 3 Centre for Advanced Reconstruction of Extremities, Sahlgrenska University Hospital/Mölndal, Mölndal, Sweden; 4 Department of Health and Rehabilitation, Institute of Neuroscience and Physiology, Sahlgrenska Academy at University of Gothenburg, Gothenburg, Sweden; 5 Hunter Medical Research Institute, University of Newcastle, Newcastle, Australia; 6 Stroke Centre West, Sahlgrenska Academy at University of Gothenburg, Gothenburg, Sweden; University of Birmingham, UNITED KINGDOM

## Abstract

**Background:**

Rehabilitative stroke interventions based on principles of multimodal stimulation have the potential to profoundly affect neuroplastic processes beyond the sub-acute phase. In order to identify important core mechanisms, there is a need to explore how interventions that combine physical, social, sensory, and cognitively challenging activities are perceived and experienced by the participants. This qualitative study, based on an interpretive interactionist perspective, explored the experiences of stroke survivors who participated in a group-based multimodal rehabilitation program based on rhythm and music.

**Methods and findings:**

Within four weeks after completion of the multimodal rehabilitation program, face-to-face semi-structured interviews were conducted on a single occasion with 15 purposively selected individuals (mean age 65, 8 men, 7 women). The interview duration was between 13 and 44 minutes. Qualitative content analysis with an inductive approach was used to analyze data. Three categories were identified, each containing several sub-categories: *To be intellectually challenged* (energy-consuming activity and coordinating multiple input and output), *Perceived therapeutic benefits* (motor skills, cognitive skills, emotional and psychological responses), and *Pros and cons with social integration* (fellowship, competing with others, and instructor characteristics). From these categories, an overall theme was derived: *The multifaceted layers of multimodal stimulation*. Enjoying music, being part of a group with peers, a skilled instructor, and being able to manage the challenging movements, were related to positive experiences. In contrast, negative experiences were associated with not being able to perform the exercises, and with group members who dominated the conversational space.

**Conclusion:**

This study shows that access to a multimodal rehabilitation program with rhythm and music as operating ingredients may contribute to positive experiences for many individuals in a late phase after stroke in terms of motor, cognitive, as well as emotional enhancements. Important components were the music, the social interaction, the challenging exercises, and the skilled instructor.

## Introduction

Long-term impairments are common in stroke survivors, including cognitive, sensory, and visual symptoms, with motor-related impairments most common, affecting 80% of stroke patients [1]. The rate of spontaneous recovery, defined as the amount of improvement in body function and activity determined solely by time [2], varies and is rarely complete [3]. It was previously believed that only limited functional gains were possible to achieve beyond six months after stroke [4], but during the last decades, a paradigm shift has occurred within the fields of neuroscience and neurorehabilitation that provides hope for improvements lasting beyond spontaneous recovery of functions [5, 6]. In order to enhance the process of functional recovery, an intense stimulation of multisensory systems is advantageous [7, 8]. Therapies that combine enriched environments and task-specific exercises (i.e., multimodal rehabilitation) appear to be more effective than single interventions with respect to improving stroke recovery [9]. To optimize effectiveness, multimodal rehabilitation interventions should engage participants in activities that combine physical, social, sensory, and cognitively challenging activities, and seem to be most favorable when carried out in a stimulating environment. Such multimodal rehabilitation programs may considerably improve the conditions for neuroplastic changes also very late in the rehabilitation process [9–11].

The vast majority of studies investigating multimodal treatment concepts are based on animal models [11]. Translating these principles into clinical settings is challenging. In a recent pilot study by Rosbergen *et al*. (2017) patients with acute stroke admitted to a stroke unit received intervention according to an enriched protocol (with the addition of stimulating items such as board games, audio books, and music). It was shown that such enrichment strategies in organized stroke care had a significant positive effect on activity levels across all activity domains [12]. The enriched protocol was further explored in a qualitative study including ten stroke survivors [13]. The participants’ experiences emphasized greater stimulation, increased socialization, and less inactivity and boredom as a result of their access to the enriched unit [13]. With the potential to enhance functional outcomes also in the late phase of stroke recovery, we designed a randomized controlled trial in order to assess the efficacy of two multimodal rehabilitation programs in a community based setting [14]. Both treatment programs included cognitively challenging exercises, integration of multi-sensory stimulus, and complex motor skill training with frequent repetition, and an increasing level of difficulty [14]. However, the main parts of the programs were quite different: rhythm and music or, alternatively, therapeutic horseback riding. The results of this trial indicated that both interventions enhanced the participants’ own perception of recovery. The effects remained for a long period: after six months, 56% in the horseback riding program experienced that their stroke recovery had further progressed, as did 43% in the rhythm and music-based program, compared with 22% of participants in the standard care group (control). In addition, objective measures confirmed functional improvements in gait, grip strength, working memory, and balance in both groups [14].

Music is now being recognized as a multimodal stimulus, as it activates many brain structures and can stimulate complex cognition and multisensory integration [15, 16]. The Rhythm and Music Therapy (R-MT) of choice, was a practitioner-led intervention created by the musician Ronnie Gardiner, specifically targeting sensorimotor and cognitive function. The R-MT was previously explored in a phenomenological study of stroke survivors, which found that the intervention helped the participants to feel connected again with their changed, unfamiliar bodies. Further, they felt a change in their ability to carry out complex activities and gained a feeling of capability. The study identified the music, the instructor, and the other group members as important facilitators during the process [17].

When evaluating the usefulness of new complex interventions, it is essential to consider both objective and subjective evaluations. To optimize the therapeutic effectiveness of such interventions, it is therefore important to also explore how recipients respond to the intervention [18]. Qualitative approaches can contribute to the evaluation of complex interventions [18] and have the potential to provide more in-depth information about phenomena under study. Qualitative methods may address some of the most pervasive concerns in rehabilitation research, such as practitioner-participant interaction, interaction between participant/environment/disease, and the subjective and lived experiences of participants [19]. Ultimately, this will serve to better inform those providing rehabilitation services, thereby benefiting the individuals who are utilizing such services. An interpretive perspective with an interactional approach can be useful for gaining a deeper understanding of the complex interactions within such a program.

The objective of the present study was to explore how R-MT, in the context of multimodal stimulation, was experienced by community-dwelling stroke survivors in a late phase after stroke.

### Ethics approval

Formal ethics approval was obtained from the Regional Ethical Review Board in Gothenburg, reference number 698–09. The study was conducted in accordance with relevant ethical guidelines including written informed consent from the participants. The study was registered on ClinicalTrials.gov, identification number NCT01372059.

## Materials and methods

In an effort to gain more insight into the experiences and meaning of participating in the R-MT program, we used a qualitative design with an inductive approach (i.e., derived from the interviews) as the general framework, following recommended guidelines for qualitative inquiry within the field of medicine [20]. The philosophical position underlying the study relied on an interpretivist orientation. The epistemological position of interpretivism is subjectivism, and the ontological position is relativism, where reality is seen as subjective and varying between persons [21]. Furthermore, the methodological approach was an interactionist perspective, where people are seen as actively creating their own development through social interaction with others [22, 23]. The reporting of the results follows the COREQ statement [24] (S1 Table).

For this interview study, a sub-population of 15 participants was purposively selected among 123 late-phase stroke survivors consecutively recruited to participate in an intervention trial between the years 2010 and 2012. All participants lived in own housing in the community and suffered from functional impairments equivalent to category 2 and 3 on the modified Rankin Scale (slight to moderate disability). The participants were randomized to either an R-MT program, a therapeutic horseback riding program, or a control group receiving the R-MT program with one-year delay. The randomization was stratified with respect to gender and hemispheric location of the stroke. In total, 41 individuals participated in the R-MT program, distributed into eight groups with between two and nine participants in each group. All groups were led by the same instructor with several years of experience from leading the R-MT. The program contained two weekly sessions during 12 weeks, each session lasting for 90 minutes including a coffee break allowing for socializing. A detailed description of the R-MT has been published elsewhere [14, 25]. The training was performed in a standardized way in all groups.

### Participant selection

A pragmatic approach was used to determine sample size because of the narrow scope of the study [26], and it was estimated *a priori* that between 15 and 20 participants would be sufficient to capture a variety of experiences instead of using data saturation. Participants formed a purposive sample, selected by one of the authors (LBK). Efforts were made to select representatives from all eight training groups to increase credibility and to capture the broadest set of experiences and perceptions. To optimize the representativeness even further, individuals with both left and right hemispheric strokes of varying ages and with different occupations (including retirees) were selected, as well as both female and male informants. Furthermore, informants with foreign origin, as well as informants with aphasia following the stroke were selected. Importantly, both individuals who reported perceived functional improvements, and individuals who perceived no functional improvements, were selected. These subjective perceptions were based on informants’ rating on a 100 mm visual analogue scale, where a 10 mm increase was considered to be a perceived improvement, and below 10 was considered to be no improvement or a deterioration of function [14] (Table 1). Informants were blinded to the dichotomization based on their rating. Seventeen individuals were invited to participate by telephone by one of the authors (GC); two individuals rejected invitation, and 15 individuals agreed to be interviewed. Both interviewers and analyzing authors were blinded to the dichotomization of informants’ rating of improvement during the process of analyzing data.

**Table 1:** Demographics of the 15 informants with stroke.

Informant	Sex	Age	mRS*	Aphasia	First-ever stroke	Perceived improvements (yes or no)
1	M	73	2	No	Yes	No
2	F	69	2	No	Yes	No
3	F	51	2	No	No	Yes
4	F	66	3	No	Yes	Yes
5	M	59	3	No	Yes	Yes
6	M	57	2	No	Yes	No
7	F	67	3	Yes	Yes	No
8	M	68	3	Yes	Yes	Yes
9	F	74	3	Yes	Yes	Yes
10	F	64	2	Yes	Yes	Yes
11	M	66	2	Yes	Yes	Yes
12	M	61	2	Yes	No	Yes
13	M	66	3	Yes	Yes	No
14	M	62	3	Yes	Yes	Yes
15	F	72	3	Yes	Yes	Yes

* modified Rankin Scale: An ordinal disability rating scale ranging from zero to 6 (0 = no symptoms). mRS grade 2 = *slight disability*: unable to carry out all previous activities but able to look after own affairs without assistance; mRS grade 3 = *Moderate disability*: requiring some help, but able to walk without assistance

### Data collection and setting

Semi-structured face-to-face interviews were conducted on a single occasion between December 2010 and January 2013, within four weeks after treatment completion. This approach suited our aim of obtaining in-depth narratives, as the interviewer could delve deeply into social and personal matters [27]. The audio recorded interviews took place at a facility adjacent to the rehabilitation unit at Sahlgrenska University Hospital and in a relaxed atmosphere to ensure confidentiality. A semi-structured question guide developed by the research team was used (Box 1). Emphasis was placed on gaining an understanding of the individuals’ experiences of the intervention. One pilot interview was conducted, and since no subsequent adjustments were made, it was included in the analysis. The initiating prompt was “Can you describe the experience of taking part in the R-MT?”. Following this, open-ended questions such as “Could you elaborate how you mean more specifically?” or “In what sense was it so?” were used to encourage informants to expand their views and experiences, and these were readily accepted by the informants. Thus, as the informants recalled their participation, the interviewer prompted them to expand on their feelings and thoughts. The term “multimodal rehabilitation” was not used during the interviews since informants were not aware of this particular design. Most interviews were conducted by GC. When interviewing informants suffering from aphasia, a highly skilled speech therapist specialized in stroke induced aphasia facilitated communication by the use of generic scaffolding and supportive conversational techniques (e.g., pictures or a timeline) [28]. One of those interviews was conducted with the verbal support of a personal assistant who was employed by the participant. This one interview was also visually recorded to support the interpretation of the verbal speech. Two of the interviews were conducted by the speech therapist alone. The speech therapist may have had previous contact with some of the participants. She did, however, not take part in the data analysis. No other personal biases were identified before the interviews. The interview duration was between 13 and 44 minutes. Both interviewers had previous experience from the R-MT program in other contexts.

**Box 1. Sample questions from the semi-structured interview guide**

- Describe with your own words how you experienced to participate in the R-MT program. What did you experience as positive? Did you experience anything negative?
- Has the R-MT program affected your physical/psychological/social abilities?
- Has the R-MT program meant anything to you in your relationships with other people?
- Has the R-MT program affected your choice of activities in life?
- Has the participation in the R-MT program affected your mood, quality of life, or confidence in the future?

### Data analysis

Each interview was transcribed verbatim into Swedish and analyzed using qualitative content analysis [29] in order to identify differences and similarities of the contents and to identify implicit meanings of the informants’ experiences. Qualitative analysis may comprise both manifest contents (close to the text), and latent contents (messages that are more distant to the text, but close to the lived experiences) [30]. This was done by a step-wise condensation from separate meaning units that all corresponded to the aim of the study into smaller codes on a manifest level [29]. The analysis process is outlined in the audit trail more in detail (S1 Appendix). In short, all interviews were initially read repeatedly and independently by GC and PP to start the process of identifying meaning units corresponding to the purpose and to acquire a good grasp of the whole. Next, the same authors compared their meaning units, and when differences appeared, they were discussed until consensus was reached. To further manage the data, all text units were transferred to a computer software system (Open Code 4.0, freely available from Umea University at http://www.phmed.umu.se/enheter/epidemiologi/forskning/open-code), and the meaning units were subsequently condensed into codes by both authors. The codes were grouped into categories with similar utterances under a descriptive concept. Further, the categories were divided into sub-categories by similarities and dissimilarities, labelled with a description of their contents. Lastly, a theme that described the latent content of the whole (implicit message) was identified by the two authors. The final set of categories and overall theme was determined by consensus in the research group during study meetings.

### Trustworthiness and reflexivity

To establish trustworthiness and scientific rigor of findings in qualitative research, the following criteria need to be addressed: credibility, transferability, dependability, confirmability, and reflexivity [21]. Efforts were made to include individuals with a wide variety of disabilities following stroke (e.g., aphasia), thereby enhancing credibility by contributing to a richer variation of the phenomena under study. To further achieve credibility, excerpts from the interviews are provided to illustrate and support the findings, as well as to make it possible for the reader to consider the relevance of the categories and sub-categories. Feedback from the participants concerning the findings would probably have added to the validity of our interpretations. Transferability was enhanced by clear and thick description of not only the experiences of the participants, but also the context, selection, data collection, process of analysis, and characteristics of the participants as well. To enhance dependability, we included a full audit trail of all steps and decisions taken during the process of analysis (S1 Appendix), a technique proposed by Lincoln & Guba [21].

With respect to reflexivity, the members of the current research team represented different professions, allowing for triangulation of the emerging process. Both authors who analyzed the data had practical experiences from the R-MT. This may have biased the findings to some extent but may also have added to their understanding of the details regarding the R-MT. Reflexivity is further discussed in the supplementary audit trail (S1 Appendix).

## Results

Demographic characteristics of the informants are provided in Table 1. Fifteen informants with a mean age of 65 years (range: 51–74) were included, and 53% were women. The time elapsed since the stroke insult was on average 2.8 years, ranging from 18 months to 4.7 years, and two of the informants had suffered from more than one stroke. According to medical records, nine informants had aphasia. On average, the interviews lasted for 27 minutes (range: 13–44). Ten informants reported perceived functional improvements (categorized as ‘yes’); and five informants reported to perceived improvement (categorized as ‘no’) (Table 1).

Data analysis identified three main categories focusing on diverse experiences from participating in the R-MT program: *To be intellectually challenged; Perceived therapeutic benefits;* and *Pros and cons with social integration*. Each category contained several sub-categories. The categories are described in detail in the next section. From these categories, an overall theme was derived, i.e.: *The multifaceted layers of multimodal stimulation*, capturing a spectrum of aspects of taking part in a multimodal sensory stimulating program including music and rhythm as main components (Table 2).

**Table 2:** Details of the qualitative content analysis with examples from meaning units, labelling codes and categories.

Main categories	Sub-categories	Example of labelling codes	Meaning units
To be intellectually challenged	Energy consuming activity	Needed to rest	I had to lie down and rest when I came back home. It was like that every single time (laughs) (P9, aphasia)
		Completely drained	I thought it was fantastic! It was great. And you became terribly tired, so you did understand that… well, I really had to use both sides of the brain. (P3)
		Exhausted afterwards	I was completely exhausted afterwards. The whole group was totally exhausted after these sessions (laughs)! (P2)
	Coordinating multiple input and output	Intellectually demanding	In the beginning I lost my concentration and lost the movements, you know. There is so much to keep track of! (P6)
		Complicated for everyone	I think this [R-MT] is probably difficult for ordinary people as well. [–––] But in contrast to us, they probably learn more quickly than we do. (P12, aphasia)
Perceived therapeutic benefits	Motor skills	Improved coordination	I had a hard time with coordination even before the stroke, to follow a rhythm, and right and left… to lift a foot and an arm, that was really hard before. But I think that it improved. (P12, aphasia)
		Arm movement	My arm felt more mobile, or smoother … (P4)
		Prompting	I walk a lot every day, and I have actually used the words when I’m out walking—boom, chic—when I move my legs, and the fact is, I may be imagining it, but it’s not that bad actually. I get less tired then. (P5)
	Cognitive skills	Improved concentration	Yes, I noticed that my ability to concentrate improved very, very much! (P6)
		Improved memory	It might be spontaneous recovery; you never know what might have happened if I hadn’t been in this project–but my own experience is that my memory has improved. (P10, aphasia)
	Emotional and psychological responses	Happier (pitch black before)	Yes, I am definitely happier now after this, it has given me joy. Before it was just pitch black. (P3)
		Mood improved and remained better	The effect remained afterwards, I feel I am in a much better mood nowadays. (P2)
Pros and cons with social integration	The fellowship	Companionship developed	We were like a family! (P14, aphasia)
		Sharing experiences from stroke	It gave me a lot to exchange experiences with other people with stroke. [–––] Well, it was interesting to hear from the others what symptoms they had. Some had problems moving around, and others–like myself–had lost their ability to speak. (P1)
		Impact of dominating participants on group dynamics	I don’t know if I enjoyed it so much, there was one person who took over all the time. He wasn’t boring, but I didn’t get to know the others at all. I might have been interested in learning about how the others coped with their strokes and so on. We could have stopped him, but it’s not that easy when you don’t know people so well. (P4)
	Competing with other	Compares oneself to others	There was one person among us who learned it so well. It was so easy for him to remember all the sounds and expressions. I thought to myself: why can’t I? I couldn’t at all reach the speed he had, he was the best in the group. The others were like me perhaps; it was difficult to see. I tried very hard, but I didn’t really make much progress. (P7)
	Instructor characteristics	Being encouraging	He helped us a lot; he said: “Next time it will work!”. He was so kind! (P14, aphasia)
		Confirmed everyone	The instructor, he made every single one of us visible by greeting everyone and saying goodbye. It made me… well, feel like someone special! And that’s important! (P6)
		Competent and experienced	He had many years of experience with these things. He had much psychology up here (points at head). A very good person. (P14, aphasia)

### To be intellectually challenged

The R-MT program contained many exercises where arms and legs had to be coordinated in complex patterns. This was considered to be very intellectually challenging. The category was built upon two sub-categories: *energy-consuming activity* and *coordinating multiple input and output*.

#### Energy-consuming activity

The R-MT program was experienced as demanding a great deal of attention by the informants, who had to be fully concentrated in order not to lose track. The informants described this as “brain draining training” with the demand that they stayed continuously focused while performing multitasking exercises. The brief opportunities to rest, sing, and listen to music in between the exercises were valued. The informants reported being completely exhausted and being in need of rest for several hours afterwards. Sometimes the feeling of brain draining forced them to carefully select between everyday activities in order to save the remaining amount of energy. It was nevertheless emphasized that it was worth the effort and the long journeys from home because of the positive experiences from the training.

*It was really exhausting, I had to lie down and rest when I came back home. It was like that every single time*. *[Informant 9]*

*It’s a fact that all training takes time. I don’t have that much time. Since I don’t have so much energy during the day, I must choose what to put the energy into. If I choose to put it into the training, there will be nothing else. There are certain things that I am obliged to do, and if I want to do something enjoyable, I might not manage to do the training*. *[Informant 12]*

#### Coordinating multiple input and output

The challenge with coordinating the exact movement to the matching symbol, while simultaneously pronouncing the correct verbal code to the steady beat of music, was seen as both fun and complicated by informants. It was experienced that the brain had to work at its full capacity, and that it was extremely difficult at the beginning of the intervention period when the method was completely new to them. After some time, it became easier and they started to notice improvements in the ability to coordinate the multiple impressions and perform the exercises.

*Well, you do understand that you use both sides of the brain. And you also notice that you get better and better at performing these exercises that we did. It wasn’t really that easy as one would think. Clearly you have to use your head, and that’s a good thing. I actually believe in the method*. *[Informant 5]*

Some informants wanted the exercises to be even more challenging as they experienced that the level of challenge was set too low for their own functional level and was more adapted to those with more severe functional disabilities. These informants found the exercises to be boring and repetitive in the long run. In contrast, others expressed that they sometimes felt inferior to their peers because the level of challenge was set too high for their own functional level.

### Perceived therapeutic benefits

The informants had various experiences related to noticeable effects from the R-MT program, including real physical improvements, but also experiences of no changes at all. It was mentioned that it was difficult to notice changes by oneself, and that only the physical assessments would show any real changes. The category was built upon three sub-categories: *motor skills*, *cognitive skills*, and *emotional and psychological responses*.

#### Motor skills

Informants told of experienced improvements in arm mobility and coordination in the paralyzed side, as well as improved gait pattern. The specific sound codes of the method, for example boom and chic (corresponding to a stomp with the right and left foot on the floor), could be used as prompting while taking a walk, enabling a firm, steady and functional gait pattern. This also made the gait more energy effective, and it was experienced as less exhausting to take a walk.

*I walk a lot every day, and I have actually used the words when I’m out walking—boom, chic—when I move my legs, and the fact is, I may be imagining it, but it’s not that bad actually. I get less tired then*. *[Informant 5]*

#### Cognitive skills

This sub-category contained examples of higher cognitive functions, such as improved memory and speech ability. The ability to focus and to concentrate for a longer time seemed to improve through the exercises. One informant reported that he was certain that he passed his exam for the driver’s license because of the improvements he had made during the intervention period. Another informant described that she had switched from listening to talking books to reading paper books again:

*I think that my ability to concentrate has improved. Because I read, I read again! I didn’t do that after the stroke, I only used audio books. But now I have started to read ordinary paper books again*. *[Informant 4]*

#### Emotional and psychological responses

In general, the informants experienced the R-MT program as inspiring, well-structured, interesting, and entertaining. Doing the exercises was experienced as invigorating and stimulating. The informants told of an increased level of energy, and in general feeling more alert. Elements of the program that stood out as emotionally rewarding were the other group members, the music, and the instructor who was found to be positive and encouraging, and who confirmed each and every individual. One informant who had suffered from a severe depression after her stroke expressed it like this:

*Yes, I am definitely happier now after this, it has given me joy. Before it was just pitch black*. *[Informant 3]*

The exercises were experienced as meaningful and inspired creativity and new initiatives. For example, two informants with aphasia told that participating in the R-MT had inspired them to begin with speech therapy on their own at home (e.g., reading out loud and practicing handwriting).

Negative emotions were also reflected upon. For example, those who had high expectations regarding improvements in cognitive and/or motor skills, but noticed no changes at all, expressed disappointment over the lack of improvements. However, most informants entered the program with an open mind without expectations, simply because they did not know what to expect. An initial skepticism about the program was also aired, but informants were mainly positively surprised by the complexity and challenges of the method, as well as by how engaging and enjoying it was:

*I think it [the R-MT] has been only positive. I was a little skeptical at the beginning, but it [the R-MT] was better than I thought*. *[Informant 1]*

### Pros and cons with social integration

This category was nonspecific to the R-MT method and more concerned with the issues of being part of an exercise group with several participants and a trained leader. The category was built upon three sub-categories: *the fellowship*, *competing with others* and *instructor characteristics*.

#### The fellowship

The informants told stories of affinity and solidarity, a bonding fellowship that developed during the intervention period while sharing experiences from living with consequences of a stroke. Meeting with other peers was of great importance for the informants’ overall well-being. They mentioned the positive aspects of a tolerant climate, increased togetherness with mutual pep, injecting each other with energy and enthusiasm, as well as mutual understanding built on a feeling of all being in the same boat.

In contrast, informants from two of the groups told of the negative impact on group dynamics resulting from a dominating participant. The dominating behavior of these participants made it more difficult for the others to bond. It was perceived by the informants that these individuals took over the conversational space. This was considered as annoying but also that it would have been impolite to interrupt these persons:

*I don’t know if I enjoyed it so much, there was one person who took over all the time. He wasn’t boring, but I didn’t get to know the others at all. I might have been interested in learning about how the others coped with their strokes and so on. We could have stopped him, but it’s not that easy when you don’t know people so well*. *[Informant 4]*

#### Competing with others

Participating in a group intervention with other people inevitably led to comparing oneself with the other participants. For some informants this was seen as a positive spurring, but for others this contributed to a feeling of inferiority, which could have led to lowered self-confidence. The informants had different functional prerequisites, with large variations within the group. It was mentioned that it would be preferable if all participants had been on the same functional level.

*There was one person among us who learned it so well. It was so easy for him to remember all the sounds and expressions. I thought to myself: why can’t I? I couldn’t at all reach the speed he had, he was the best in the group. The others were like me perhaps; it was difficult to see. I tried very hard, but I didn’t really make much progress*. *[Informant 7]*

#### Instructor attributes

The instructor played an important role for the well-being of the informants. Important aspects were that the instructor was perceived as calm, competent, and experienced, encouraging, and pedagogical. By confirming each and every one of the individuals he created a sensation of ‘being someone’, strengthening the self-confidence of the participants. The instructor also frequently gave positive feedback and kept track on those who could not keep up.

## Discussion

We conducted a qualitative study to explore stroke survivor’s experiences from a group-based multimodal rehabilitation program based on rhythm and music in a community setting. The informants’ stories revealed a spectrum of aspects. The findings suggest that access to a multimodal rehabilitation program based on rhythm and music may contribute to therapeutic benefits for many individuals in a late phase after stroke in terms of motor, cognitive, as well as emotional enhancements. The experienced motor and cognitive improvements correspond to objective findings, confirming that the perception of recovery was higher among R-MT participants compared to controls on a group level [14].

Multimodal interventions in socially interactive contexts have the potential to improve functional abilities in individuals recovering from stroke [9, 11]. In order to drive activity-dependent neuroplasticity, it is crucial that the training is perceived as functionally relevant to the individual [31]. If the training is experienced as rewarding and stimulating, it most likely could increase neural plasticity. On the contrary, a training perceived as non-rewarding would potentially lead to less improvement. Accordingly, it is argued that simply repeating exercises will not induce reorganization after brain damage [31]. In this study, the R-MT was in general experienced as meaningful, enjoyable, and invigorating, but some informants also reflected upon non-rewarding experiences with the potential to disrupt functional gains. For example, being too able as compared to others could make the exercises boring and repetitive without challenge while being too disabled with slow progress could lead to a feeling of inferiority towards the other participants. Positive experiences were gained from the social interaction, the challenging exercises, the musical components, a real sense of functional progress, and the presence of a qualified professional. The findings are in line with Thornberg *et al*., who identified the music, the instructor, and the other group members as facilitators within the same R-MT program [17]. Enjoyable activities leading to positive experiences are supposedly what would be required of a multimodal sensory stimulating program with the purpose to enhance neuroplastic processes in a late phase after stroke [6]. During the last decade, researchers have paid particular attention to the neurophysiological background to musical processing, especially with respect to the involvement of emotion, mood, and relevant brain networks involved in these processes [32]. Positive affective responses with sensory pleasures are strongly related to these networks via dopaminergic pathways [32]. Promoting positive mood change and motivating participants to initiate and continue with rehabilitation long-term is often a challenge to clinicians, and the use of the R-MT may be of assistance in this process, as well as boosting self-esteem.

The informants in this study spoke of the meaning of being part of a social group. Belonging to a group of peers has also been found beneficial in other activity-based studies involving stroke survivors [17, 33]. In addition, studies on mice have shown that social interaction plays a critical role for neurogenesis and recovery after stroke [34], why this is an important addition to multimodal activities. With respect to the social interaction, therapeutic relationships could focus on aspects such as group cohesion. Group cohesion in the physiotherapy context has been defined as a construct that includes the following dimensions: individual attraction to the group, individual attraction to the social dimensions of the group, perception of integration of the group around its task, and perception of integration of the group around social bonding within the group [35]. Group atmosphere, described as the therapeutic environment perceived by participants in a therapy group, is a group level phenomenon, meaning that within the group, each participant might perceive the atmosphere of the group differently [36]. Informants in the present study confirmed friendship and bonding to be important sources of positive experiences, but there was also of a sense of competition. For some, this was a positive and stimulating experience, but for others a negative and stressful experience. Introducing competition inducing factors in group activities may generate individual rewarding behaviour at the cost of group cohesion and added stress [37]. Moreover, the presence of one dominant individual who took up a large amount of conversational space made the social interaction difficult for individuals who were less persistent. This may be a personality trait [38] or a consequence of the stroke affecting social skills one is not aware of [39]. It is probable that this phenomenon was more apparent during the 30-minute coffee break, but this was not explored.

In agreement with a previous study on the R-MT program, the instructor was an important part of the positive experience [17]. Attributes of the instructor as being pedagogical, calm, competent, encouraging, and confirming were emphasized by the informants in this study. The attribute of the group leader concerning the ability to develop and maintain a positive learning climate has been found important for motivation and compliance [40]. Belonging to a new group does not automatically lead to developing one’s social identity and needs to be carefully nurtured through the actions of the group leader [41].

Multimodal interventions might be favorable to introduce in various settings, and be incorporated into different rehabilitation pathways, as the stroke survivors needs and willingness to participate are likely to change over time [42]. The positive experiences of multimodal stimulation may be initiated already during hospital stay, where an enriched protocol was found to be beneficial early in the rehabilitation process [12, 13]. In the community reintegration phase, meaningful and long-term activities are warranted to counteract boredom that otherwise may lead to depression and worsening of function, affect, health status, and quality of life [43]. Therefore, it might be favorable to introduce such interventions in various settings. It is, however, important to allow individuals to take an active part in the decision-making process based on individual needs, preferences, and values regardless of phase of recovery [44]. It may therefore be of great importance to offer different forms of activities based on principles of multimodal stimulation. The R-MT program may be highly appreciated by many individuals, but not by all. The experiences and perceptions may be of best benefit if the participant enjoys music, enjoys being part of a social group with peers on a similar functional level, and if the exercises are challenging on a moderate level. There may also be reason to match the participants with peers on a similar functional level in order to optimize the positive and enjoyable experiences and to reduce the risk for competition momentum. However, it might also be argued that individuals who have isolated themselves socially after the stroke may benefit as well. More knowledge about such aspects are warranted.

In conclusion, the findings of this study suggest that access to a multimodal rehabilitation program with rhythm and music as key components may contribute to positive experiences for many individuals in a late phase after stroke in terms of motor, cognitive, as well as emotional enhancements. The R-MT was in general experienced as meaningful and enjoyable, and components of importance were identified as the musical element, the social interaction, the challenging exercises, and the skilled instructor.

### Methodological considerations

The credibility and richness of data was further strengthened by including individuals with a wide variety of disabilities following stroke, including severe aphasia. The 15 informants were selected based on the assumption of a rich diversity and ability to express experiences and perceptions from the perspective of a late phase after stroke. A major strength of this study was that we included participants who did not perceive functional gains from taking part in the R-MT program. This approach was deliberately used in order to capture negative, as well as positive, aspects of the participation experience. Another strength is that we selected at least one representative from every group out of eight groups.

This study was conducted with a relatively small number of participants, which limits the transferability and therefore, the findings are not transferable to the general population. Furthermore, this study examines the experience of stroke survivors with slight or moderate disability (category 2 and 3 on the modified Rankin Scale), and therefore the findings are not generalizable to stroke survivors with more severe disability (category 4), or to other neurological disorders. With respect to external validity, a limitation was that the participants’ experiences are related to Swedish societal contexts. This needs to be considered when the knowledge is transferred to other cultural contexts. We believe, however, that this program may provide rich experiences and have a profound impact on stroke survivors’ emotional and physical state regardless of geographical area. For example, the use of rhythm and music was appreciated by the informants in this study. Music is a universal stimulus which makes it easy to adapt to other cultures and useful in stroke rehabilitation [45], although it is possible that the cultural attitudes towards rhythm and music may vary between countries. In addition, the R-MT program requires very little equipment; therefore, this multimodal rehabilitation program can be considered to be inexpensive and easy-to-conduct, especially when delivered in groups of several participants.

Future studies should be performed in order to reproduce the results from this study, as well as investigate whether individuals with different neurological disorders, or with more severe stroke disability, have similar or divergent experiences from participating in the R-MT program.

## Supporting information

S1 Table Reporting according to the COREQ criteria. (DOCX)

S1 Appendix Audit trail. (DOCX)
