# Supplementary material for: Experiences from a multimodal rhythm and music-based rehabilitation program in late phase of stroke recovery – A qualitative study
Source: PLoS One. 2018 Sep 18;13(9):e0204215. doi: 10.1371/journal.pone.0204215 (PMC6143265; doi:10.1371/journal.pone.0204215)
Supplement: S1 Table — (DOCX) [file pone.0204215.s001.docx]

**S1. COREQ 32-item checklist**

| **No. Item** | **Guide questions/description** | **Comments** |
| --- | --- | --- |
| **Domain 1: Research team and reflexivity** |  |  |
| *Personal characteristics* |  |  |
| 1. Interviewer/facilitator | Which author/s conducted the interview? | GC with support of a speech therapist |
| 2. Credentials | What were the researcher’s credentials? *e.g., PhD, MD* | 2 MD and Professors; all PhD |
| 3. Occupation | What was their occupation at the time of the study? | Research Fellows |
| 4. Gender | Was the researcher male or female? | 3 female/2 male |
| 5. Experience and training | What experience or training did the researchers have? | > 20 years of qualitative research |
| *Relationship with participants* |  |  |
| 6. Relationship established | Was a relationship established  prior to study commencement? | Possibly by the speech therapist |
| 7. Participant knowledge of the interviewer | What did the participants know about the researcher? *e.g., personal goals, reasons for doing the research* | Broad outlines given |
| 8. Interviewer characteristics | What characteristics were reported about the interviewer/facilitator? *E.g.,* *bias,* *assumptions, reasons and interests* *in the research topic* | Experience from the R-MT |
| **Domain 2: study design** |  |  |
| *Theoretical framework* |  |  |
| 9. Methodological orientation and Theory | What methodological orientation was stated to underpin the study? *e.g. grounded theory, discourse* *analysis, ethnography,* *phenomenology, content analysis* | Interpretive inter­actionism (content analysis was used for data analysis) |
| *Participant selection* |  |  |
| 10. Sampling | How were participants selected? *e.g. purposive, convenience,* *consecutive, snowball* | Purposive |
| 11. Method of approach | How were participants approached? e*.g. face‐to‐face,* *telephone, mail, email* | Telephone |
| 12. Sample size | How many participants were in the study? | 15 |
| 13. Non-participation | How many people refused to participate or dropped out? Reasons? | Two individuals refused to participate, no reasons given |
| *Setting* |  |  |
| 14. Setting of data collection | Where was the data collected? *e.g. home, clinic, workplace* | At a rehabilitation unit |
| 15. Presence of non-participants | Was anyone else present besides the participants and researchers? | Yes, one personal assistant and one speech therapist |
| 16. Description of sample | What are the important characteristics of the sample? *e.g.* *demographic data, date* | People with stroke in a late phase of re­co­very |
| *Data collection* |  |  |
| 17. Interview guide | Were questions, prompts, guides provided by the authors? Was it pilot tested? | Semi-structured interviews. Pilot tested |
| 18. Repeat interviews | Were repeat interviews carried out? | No |
| 19. Audio/visual recording | Did the researchers use audio or visual recording to collect the data? | Both |
| 20. Field notes | Were field notes made during and/or after the interview or focus group? | No. |
| 21. Duration | What was the duration of the  interviews or focus group? | Between 13 and 44 minutes |
| 22 Data saturation | Was data saturation discussed? | Yes |
| 23. Transcripts returned | Were transcripts returned to parti­ci­pants for comment and/or correc­tion? | No member checks were performed. |
| **Domain 3: analysis and findings** |  |  |
| *Data analysis* |  |  |
| 24. Number of data coders | How many data coders coded the data? | Two (PP and GC) |
| 25. Description of the coding tree | Did authors provide a description of the coding tree? | No. |
| 26. Derivation of themes | Were themes identified in advance  or derived from the data? | Derived from the data, i.e., inductive |
| 27. Software | What software, if applicable, was used to manage the data? | OpenCode |
| 28. Participant checking | Did participants provide feedback on the findings? | No |
| *Reporting* |  |  |
| 29. Quotations presented | Was participant quotations presen­ted to illustrate the themes/findings? Was each quotation identified? *e.g. participant number* | Yes |
| 30. Data and findings consistent | Was there consistency between the data presented and the findings? | Yes |
| 31. Clarity of major themes | Were major themes clearly presented in the findings? | Yes |
| 32. Clarity of minor themes | Is there a description of diverse cases or discussion of minor themes? | Yes |
