# Supplementary material for: Experiences from a multimodal rhythm and music-based rehabilitation program in late phase of stroke recovery – A qualitative study
Source: PLoS One. 2018 Sep 18;13(9):e0204215. doi: 10.1371/journal.pone.0204215 (PMC6143265; doi:10.1371/journal.pone.0204215)
Supplement: S1 Appendix — (DOCX) [file pone.0204215.s002.docx]

# Background and study context

Semi-structured interviews were conducted in the context of a randomised controlled trial (RCT) entitled ‘Long-term improvements after multimodal rehabilitation in late phase after stroke’.

## decisions taken prior to the present study

The philosophical position underlying this study relied on an interpretivist orientation, as opposed to the positivist orientation driving the RCT. The ontological position of interpretivism is relativism, which is the view that reality is subjective and differs among people (Guba & Lincoln, 1994). An in­ter­actionist perspective was adopted as the methodological approach. From this perspective, people are seen as actively participating in the creation of their own development through interaction with the social world. This relational approach deals with how people shape their realities through in­ter­action and interplay with other people in relation to a social context (Mead, 1962; Blumer 1986).

The interpretivist approach is based on a naturalistic approach to data collection. It was therefore decided to use semi-structured face-to-face interviews as data source.

These philosophical and theoretical standpoints form the basis for how we chose to view and under­stand the social worlds and interactions within the intervention groups.

## Research team and reflexivity

The research team consisted of five researchers with different professional back­grounds and exper­tise from different fields of health care, and this was expected to broaden the inter­pretation of the analysis. GC is a PhD and occupational therapist; LKB is a PhD and physio­therapist; PP is a PhD and physiotherapist as well as a trained medical secretary; MN is a university professor and physician; and CB is also a university pro­fes­sor and physician. All re­sear­chers have many years of experience from working with stroke rehabilitation in different settings as well as different cities and countries.

GC and PP have formal training and several years of experience in qualitative me­thods. The other co-authors have no formal training in qualitative methods but have co-authored several studies with qua­litative methodology. Throughout the analysis process, the research team recognized that pre-understanding, values and assumptions does affect the interpretation of the data. For example, there was a pre-understanding within the research team that multimodal rehabilitation may be bene­ficial in a late phase of recovery. This might have had an impact on the interpretation and ana­lysis of the data. CG and PP both have personal experiences from taking part in the rhythm-and-music based (R-MT) intervention, which may have biased their analysis of the data in a positive direc­tion. Prior to the study, no relationships between the interviewer GC and the participants were esta­blished. A speech therapist was employed to support interviews with people suffering from aphasia. This speech therapist may have had previous contact with some of the participants. She did, however, not take part in the data analysis. No other personal biases were identified before the interviews.

### Sample size

A pragmatic approach to the determination of sample size was used instead of using data saturation because of the rather narrow research question, which was to explore participants’ experiences of the R-MT. It was esti­ma­ted *a priori* that between 15 and 20 participants would be suffi­ci­ent to cap­ture a variety of expe­ri­en­ces.

### Participant recruitment

One male participant died during the intervention period. During the first physical follow-up assess­ments, the remaining 40 participants from the R-MT group were asked if they would agree to be inter­viewed within four weeks after the intervention, as well as in groups after six months. The moti­vation given was that questionnaires and physical assessments would not suffi­ci­ently capture their personal experiences and perceptions of taking part in the multimodal inter­ven­tions. All partici­pants agreed to be contacted.

### Developing the interview schedule

The research team discussed possible main questions and prompts that could be used in order to identify participants’ perceptions and experiences from taking part in the group-based active inter­ventions. Emphasis was placed on gaining an understanding of the individuals’ experiences of the intervention. It was also emphasised that the questions should be neutral rather than value-laden or leading. The first draft of the interview questions was considered to be too explicit and linked to specific assumptions. The second draft of the semi-structured interview schedule was then constructed, with the follo­w­ing questions:

- Describe in your own words your experience of participating in the R-MT group. Positive or negative experiences?
- Has this participation had any effect on your physical, psychological or social abilities?
- Has participation meant something for you in your contact with other people? In connection with R-MT group? In other contexts?
- Has participation affected your activity performance in life in general?
- Has participation affected your life situation in general?
- Has participation affected your mood, quality of life, and beliefs about the future?

Prompts were given to encourage participants to expand upon their feelings and thoughts, such as “In what way was it so?”; or “Could you elaborate how you mean more specifically?”. One pilot interview was conducted, which was later included in the analysis because no changes were believed necessary.

# audit trail for the present study

The following audit trail solely concerns the research procedure for the individual interviews con­duc­ted with participants from the R-MT groups, and is therefore based on the face-to-face interviews within four weeks after the intervention. The research question for the present study was to explore stroke survivors’ experiences from taking part in a group-based multimodal R-MT intervention.

## Sample selection and participant recruitment

One of the authors (LBK) purposively selected 17 potential interviewees from the list of the 40 par­ticipants from the R-MT group. Participants from all eight intervention groups were selected to cap­ture the broadest set of experiences and perceptions. To optimize representativeness, individuals with both left and right hemispheric strokes were selected, as well as both men and women, par­ti­ci­pants with various ages and work experiences (including retirees), and individuals from other count­ries as well as individuals with aphasia following the stroke. In addition, efforts were made to include individuals who had reported both perceived functional improvements, and those who had reported no functional improvements. These perceptions were based upon participants’ rating on a 100 mm visual analogue scale, where 10 mm increase from baseline was considered to be a subjectively per­ceived improvement, and below 10 mm was considered to be no improvement or a deterioration of functional abilities. A list of 17 individuals was thus delivered to GC, who contacted all individuals by telephone with an invitation to be interviewed. Two individuals kindly rejected the invitation with­out lea­ving explanations, and 15 agreed to be interviewed. No monetary incentives were involved.

## Data collection

Interviews were conducted on a single occasion between December 2010 and January 2013 at a re­ha­bilitation facility at Sahlgrenska University Hospital in Gothenburg, Sweden. The setting for the in­terviews was chosen to ensure confidentiality in a relaxed atmo­sphere. One pilot interview was con­ducted, and no adjustments were made to the question guide. The pilot interview was therefore in­cluded in the unit of analysis. Fourteen further participants underwent face-to-face interviews with GC, or a speech therapist, or both together. The speech the­ra­pist was there to support the inter­pre­tation of the participants with aphasia, and conducted two of the interviews on her own. The inter­views lasted 13–44 minutes. The interviews were audio recorded with a digital audio recor­der.

## Data transcription

The interviews were transcribed verbatim in Swedish between June 2012 and July 2014 by a person external to the research group who had no influ­ence over the analysis process. One interview was tran­scribed by the speech therapist because of the participants’ severe aphasia. Utterances such as ‘mm’, ‘eee’, emphasised words, laughter, or pauses were included within the transcripts. No mem­ber checks were con­ducted for comment or correction after the data transcription.

The transcripts were verified for accuracy by PP by cross-checking the tran­s­­c­ripts with the audio recordings between July 2015 and March, 2016. The texts were somewhat shortened in that utte­ran­­ces such as ‘eee’ or ‘mm’ were removed from the transcribed texts if they were made by the interviewer, and did not add to the contents. All transcripts were anonymised by a unique number prior to the analysis (P1 to P15).

## data analysis

Qualitative content analysis (QCA) was used as analytical method as descri­bed by Graneheim and Lundman (2004 & 2017). This analysis approach is appro­pr­iate when the aim is to focus on the sub­jects as well as on the contextual meanings. The emphasis in QCA lies on describing variations, e.g. simi­­la­ri­ties and dif­fe­ren­ces within parts of the text. QCA may comprise both descriptions of the mani­fest contents (close to the text), as well as of the latent messages (more distant from the text, but close to the lived experien­ces). We have mainly described the manifest contents in this study, but this also requires some degree of interpreta­tion. The corrected text files were considered as the unit of analysis.

We chose an inductive approach when analysing the data, given the research question. With this app­roach, little or no predetermined theory, structure, or framework is used to analyse data. This is recommended when there is little former knowledge about the phenomenon of interest. Thus, the themes were derived from the data and not on the basis of previous knowledge or theories.

All team members read the interviews, but GC and PP conducted the preliminary analysis of the col­lec­ted data. Consensus was reached during several team meetings. The process of coding the mate­rial and deriving the final categories was done in a step-wise process between March and June, 2016.

**Step 1**. All interviews were initially read independently by PP and GC to acquire a good grasp of the whole, and to start the pro­cess of identifying meaning units. Meaning units are based on a few words or sentences that correspond only to the research question, i.e., how the R-MT was expe­ri­enced. Sometimes the participants spoke about for the study irrelevant things, such as a pleasant journey, and those sections were ignored.

**Step 2.** During four personal meetings, GC and PP compared their meaning units, side by side. When differences appeared, the meaning units were discussed until sufficient agreement was reached. During this process of comparing meaning units, asso­ciations were made, and ideas and thoughts were noted in the margin of the transcribed texts.

**Step 3.** Next, the text units (i.e., raw data) were formatted into txt. -files and transferred to a com­puter software system (Open Code 4.0, freely available from Umea University at <http://www.phmed.umu.se/enheter/epidemiologi/forskning/open-code>) to be further processed.

**Step 4.** All Swedish meaning units were condensed into labelling codes by GC and PP authors toge­ther. A labelling code is a dense description that describes the contents of the meaning unit with 1–5 words. The codes are very close to the text not to lose the core content of the unit. The codes were marked when the participant had aphasia. Table 1 shows examples of meaning units and their labelling codes (translated into English) (Table 1).

| Table 1. Examples of meaning units and their labelling codes. P = Participant; I = Interviewer | | |
| --- | --- | --- |
| **Code** | **Meaning unit** | **Labelling code(s)** |
| P1 | *“Well, it was interesting to hear from the others what symptoms they had. Some had problems moving around, and others – like myself – had lost their ability to speak.”* | - exchanging experiences with peers |
| P5 | *“The instructor, he explains: “Now you do it like this”, and then he keeps track of everyone. He immediately notices when someone is unable to follow the exercises.”* | - the instructor explains good  - the instructor keeps track |
| P11 | *“These twelve weeks went by so fast. I think the training was great! But I think it was a bit too repetitive in the end!”* | - great training – time went fast  - repetitive in the end (R-MT) |
| P13 | *[I: “These words, were they difficult? Boom, chic…”]*  *“Eeeh, no, no, I don’t know… what to say… No, maybe, I don’t know, not so much.”* | - not so difficult (R-MT)  (p. with aphasia) |
| P15 | *[I: “These words, were they difficult? Boom, chic…”] “Mm, yes (points at his arm).” [I: “You mean that you have difficulties to move your right arm?”] “Difficult! This was easy (lifts left arm), and this (stomps with both feet), but this was difficult! (points at right arm).”* | - difficult to move paralysed arm (R-MT) (p. with aphasia) |
|  |  |  |

**Step 5.** The codes were printed on paper and cut into pieces (appr. 330 codes). The codes were there­­­­after grouped according to underlying patterns that indicated similarity in contents and label­led with a primary headline describing their con­tents (Figure 1). This was done on a whiteboard with the advantage of freely moving the codes. The process of grouping codes was conducted through several meetings between GC and PP, while continuously going back to the original texts.


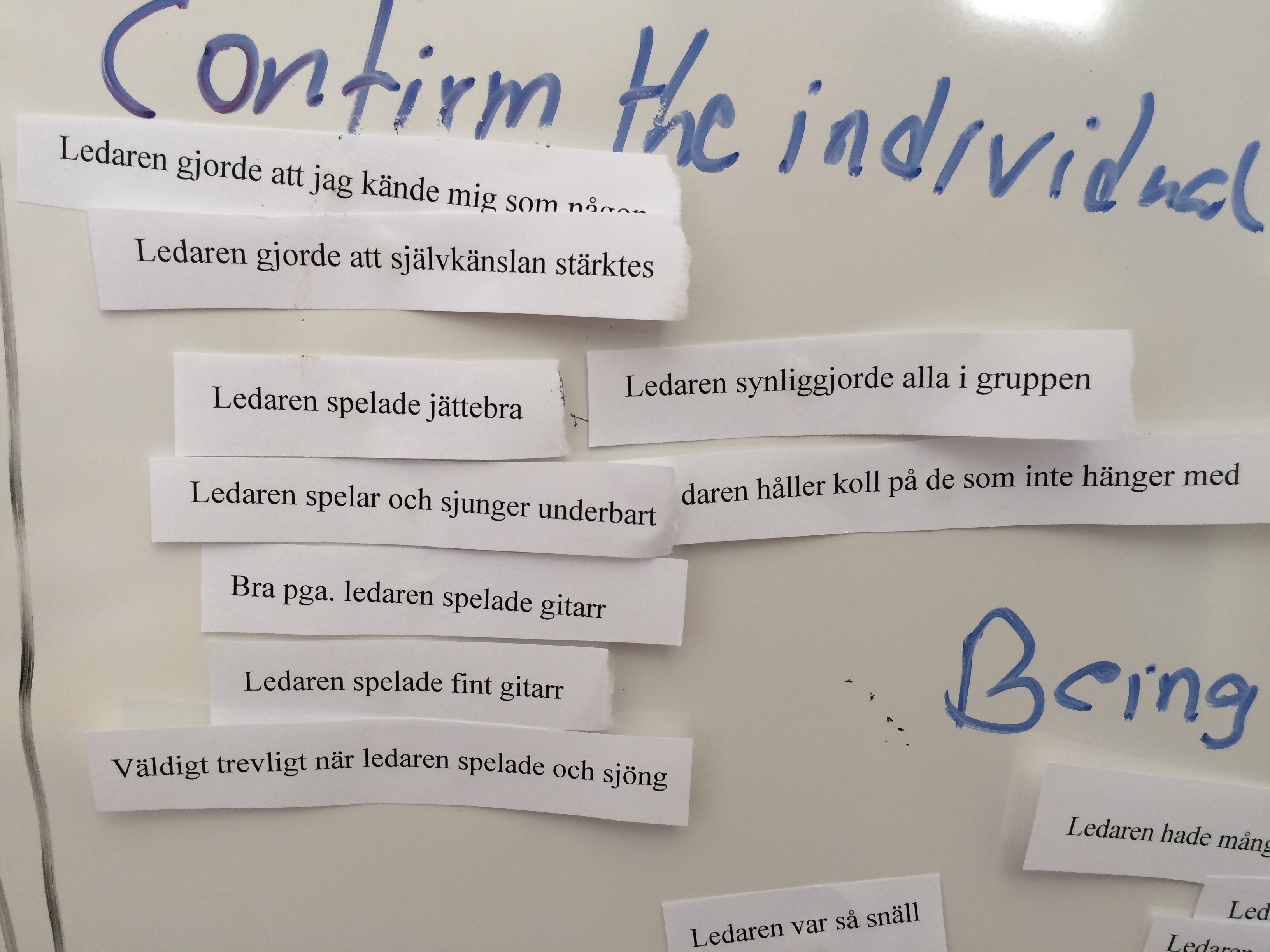

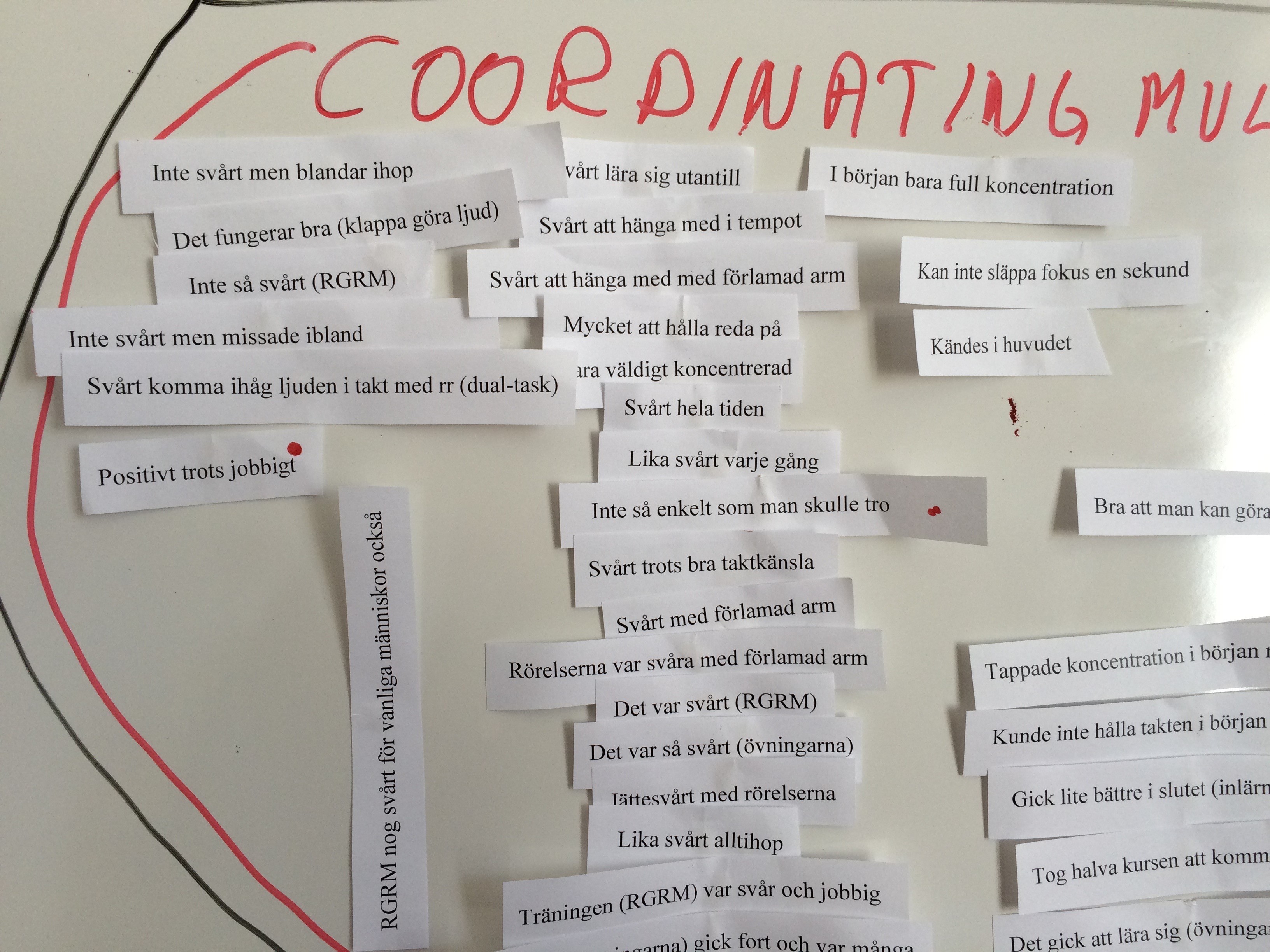


Figure 1**.** Example of how the Swedish codes were moved around on a whiteboard in order to find underlying patterns

In April, 2016, initial categories started to take form:

**The social context of participating in a group intervention**:
- fellowship (comradeship; sharing experiences; impact of dominating participant on group dynamic)
- competing with others (spurring each other; lowering self-efficacy)
- instructor characteristics (being encouraging; confirming the individual; being competent)

**Experiences from taking part in a multimodal sensory stimulating activity**:
- effort takes all energy (energy consuming; completely drained)
- the challenge of coordination multiple input and output (intellectually demanding)

**Experienced impacts**:
- motor behaviour (coordination; arm movement; prompting)
- cognitive function (memory; ability to focus and concentrate)
- emotional and psychological effects (happier; mood regulating; more alert)
- fulfilment of expectations (better than one had expected)

In June, 2016 the categories had evolved. This new set of categories were presented along with the codes to the other three team members (LBK, MN and CB) (Table 2). Their role was to consider the ana­lysis in relationship to the transcribed data and to comment on the labelling of categories as well as the appro­priateness of the categories and sub-categories, respectively. Notes were continuously taken during the meeting. During this meeting, a first suggestion for a general theme was discussed: “lived experiences from a stroke therapy program”. Following this discussion, further analysis was conducted by GC and PP, while re-reading the transcriptions.

**Step 6**. A second set of categories and sub-categories were presented to the research team. The final set of categories was thoroughly discussed, and determined by consensus.

**Step 7**. Manuscript was written by PP and GC and edited by LKB, MN and CB, reporting followed the recommendations of COREQ (Tong et al).

| Table 2. First suggestion for categories, sub-categories and detailed contents of all labelling codes – long form presented to the other authors. Some of the labelling codes have been kept in Swedish in their raw form to enhance transparency. | | |
| --- | --- | --- |
| Main categories | Sub-categories | Labelling codes |
| The social context of participating in a group intervention | The fellowship | Camaraderie (kamratskap, gemenskap, samhörighet) |
|  |  | Sharing experiences from stroke |
|  |  | Impact of dominating participants on group dynamics |
|  | Competing with others | Spurring each other |
|  |  | Lowering self-confidence by feeling inferior |
|  | Instructor characteristics | Being encouraging (humor, energisk, peppande, stärker självförtroende, noga med träna balans) |
|  |  | Confirming the individual (kände mig som någon, ger feedback, synlig­gör alla, o håller koll på de som inte hänger med) |
|  |  | Being competent (erfaren, pedagogisk, förklarar bra, möjliggör träningen, lugn, trevlig, duktig) |
|  |  |  |
| Experiences from a multimodal sensory-stimulating activity | Brain training and brain draining exercises | Need to rest, completely drained, exhausted |
|  | The challenge of coordinating multiple  input and output | Intellectually demanding, fun but complicated |
|  |  |  |
| Perceived therapeutic benefits | Motor behavior | Coordination, arm movement, prompting |
|  | Cognitive function | Memory, ability to focus and concentrate |
|  | Emotional and psychological effects | Happier, mood regulating, more alert |
|  | New inspiration | Började läsa böcker, och läsa  Ville prova nya saker |
